# *Bacillus pumilus* Cyanide Dihydratase Mutants with Higher Catalytic Activity

**DOI:** 10.3389/fmicb.2016.01264

**Published:** 2016-08-12

**Authors:** Mary A. Crum, B. Trevor Sewell, Michael J. Benedik

**Affiliations:** ^1^Department of Biology, Texas A&M University, College StationTX, USA; ^2^Structural Biology Research Unit, Department of Integrative Biomedical Sciences, Institute of Infectious Disease and Molecular Medicine, University of Cape TownCape Town, South Africa

**Keywords:** cyanide dihydratase, protein engineering, nitrilase, bioremediation, protein stability

## Abstract

Cyanide degrading nitrilases are noted for their potential to detoxify industrial wastewater contaminated with cyanide. However, such application would benefit from an improvement to characteristics such as their catalytic activity and stability. Following error-prone PCR for random mutagenesis, several cyanide dihydratase mutants from *Bacillus pumilus* were isolated based on improved catalysis. Four point mutations, K93R, D172N, A202T, and E327K were characterized and their effects on kinetics, thermostability and pH tolerance were studied. K93R and D172N increased the enzyme’s thermostability whereas E327K mutation had a less pronounced effect on stability. The D172N mutation also increased the affinity of the enzyme for its substrate at pH 7.7 but lowered its *k*_cat_. However, the A202T mutation, located in the dimerization or the A surface, destabilized the protein and abolished its activity. No significant effect on activity at alkaline pH was observed for any of the purified mutants. These mutations help confirm the model of CynD and are discussed in the context of the protein–protein interfaces leading to the protein quaternary structure.

## Introduction

Cyanide has been used for decades by the mining industry (La Brooy et al., 1994) owing to its high affinity for metals; cyanide binds and extracts metal ions from the ore by carrying them into solution. The subsequent discharge of cyanide-contaminated wastewaters into the environment is problematic because of high toxicity. Removal or transformation of the cyanide is necessary to reduce its residual concentration to levels that meet environmental standards for discharge. Traditional decontamination methods involve oxidation of cyanide via hydrogen peroxide, chlorine, or sulfur dioxide (Knorre et al., 1984; Devuyst et al., 1989). Even though these are successful in lowering the concentration of cyanide, they have several disadvantages, notably the cost of reagents, the special handling needed, and the production of adverse byproducts.

These limitations lead to significant interest in the potential for the biodegradation of cyanide using microbial or enzymatic methods. One significant success in this respect has been the biological treatment of cyanide solution in the mining environment, using rotating biological vessels (Baxter and Cummings, 2006) incorporating a microbial consortium for cyanide transformation.

An alternative approach is direct enzymatic degradation using cyanide degrading enzymes such as nitrilases. Cyanide dihydratases (CynDs) catalyze the conversion of cyanide into ammonia and formate while cyanide hydratases (CHTs) transform cyanide into formamide. Even though a variety of other enzymes also can act on cyanide, CynDs and CHTs do not require cofactors or secondary substrates, favoring them for industrial applications. Few organisms produce CynD, the best characterized are those of *Bacillus pumilus* and *Pseudomonas stutzeri* (Meyers et al., 1993; Watanabe et al., 1998) while numerous fungi, such as *Neurospora crassa, Aspergillus nidulans*, and *Gloeocercospora sorghi*, produce CHT (Basile et al., 2008).

Industrial applications of these enzymes would benefit from improved characteristics such as catalytic efficiency and stability. Numerous reports describing the successful improvement of enzymes have been published, as have a number of patents describing the biotechnological applications of these improved enzymes (DeSantis et al., 2003; Jaeger and Eggert, 2004).

One of the biggest issues with enzymatic treatment is that cyanide-containing waste-water is routinely kept at elevated pH, such as pH 11 or above. Cyanide is volatile and forms hydrogen cyanide gas at neutral pH, hence solutions are kept quite alkaline to limit formation of hydrogen cyanide. We previously applied random mutagenesis to the generation of pH tolerant mutants of CynD from *B. pumilus* (Wang et al., 2012). The parent enzyme had an optimal activity at pH 7.7 but rapidly lost activity above pH 8.5 (Jandhyala et al., 2005) whereas mutants exhibited an increase in stability relative to the wild type and *Escherichia coli* cultures expressing the mutants were capable of degrading cyanide at pH 10. Random mutagenesis followed by screening for mutants showing an enhancement in enzyme properties will, together with the recently published X-ray structure of a CynD homolog (Zhang et al., 2014), assist in the understanding the mechanism of these enzymes and will ultimately enable the rational design of enzymes with desired properties.

Here we describe the isolation and properties of 3 CynD mutants discovered following error-prone PCR that showed higher catalytic activity *in vivo* when compared to the wild type enzyme. We characterized the effect of the mutation(s) on the kinetics, stability and pH activity of each enzyme and attempt to rationalize the effects on the basis of the recent nitrilase structure (Zhang et al., 2014). The point mutation K93R increased the enzyme’s thermostability by several fold whereas E327K and D172 mutation had lesser but still notable effect on improving stability. The D172N variant also increased the affinity of the enzyme for its substrate at pH 7.7 suggesting a contribution of that residue to the active site, D172N also lowers the *k*_cat_. Another mutation, A202T, that was co-isolated with D172N, by itself rendered the enzyme inactive. No significant effect on activity at alkaline pH was observed for any of the purified mutants.

## Materials and Methods

### Culture Media and Reagents

All strains were grown in LB broth (Lysogeny Broth, Lennox). Antibiotics were used at concentrations of 100 μg/ml for ampicillin or 25 μg/ml for kanamycin or chloramphenicol as needed.

### Bacterial Strains and Plasmids

*Escherichia coli* strain MB3436 [Δ*endA thiA hsdR17 supE44 lacI^q^ZΔM15*] was used as host strain for DNA manipulations and strain MB1837 [BL21 (DE3) pLysS] was used for protein expression. The *E. coli* strain MB4091 [DH10B (pKD46)] was used as the recipient for *in vivo* recombination and MB4105 [*supE thi-1 ΔendA Δ(lac-proAB) Δ(mcrB-hsdSM)5*/F′ [*traD36 proAB^+^ lacI^q^ lacZΔM15*] was used as host for the positive selection vector pMB4105 (Abou-Nader and Benedik, 2010). The plasmids used are described in Supplementary Table S1.

### Error-prone PCR

The plasmid template for error prone PCR (EP-PCR) was pMB3980, derived from pBluescript II KS+ (Stratagene) containing the *CynD* gene encoding the *B. pumilus* C1 CynD with an N-terminal his-tag. PCR reactions were performed in 50 μl reactions containing 25 μl of Taq 2X Master Mix (New England Biolabs) with 150 ng of DNA template, 100 ng of each PCR primer, 0.2 mM MnCl_2_, and 2.5 mM MgCl_2_ added to the reaction mixture and adjusted to 50 μl with MQH_2_O. The reaction conditions were as follows: 25 cycles of 95°C for 30 s, 55°C for 60 s, and 72°C for 90 s. The PCR primers used were the -60M13 universal sequencing primers with the following sequences: -60M13F (5′-GCGAA AGGGG GATGT GCTGC AAGG); -60M13R (5′-CACTT TATGC TTCCG GCTCG TATG). The PCR products were ethanol precipitated, resuspended in water and stored at -20°C. DNA concentration was determined by measuring the absorbance at 260nm using a Nanodrop ND-1000 spectrophotometer.

### Mutant Library Construction

*In vivo* cloning (Abou-Nader and Benedik, 2010) was used to clone PCR fragments generated from EP-PCR into the positive selection vector pMB4105. PCR product (1pmole) were mixed with 0.25 pmole of linearized vector and transformed into electro-competent cells MB4091[DH10B (pKD46)] made from cells grown in LB broth at 30°C to an OD_600_ of 0.3 with 0.1% arabinose added for the final 1 h following the protocol described in Abou-Nader and Benedik (Abou-Nader and Benedik, 2010). After the electroporation, 1 ml of LB broth was added and cells were incubated for 30 min at 37°C on a roller drum for recovery. 100 μl of cells were then spread on LB plates supplemented with 25 μg/ml chloramphenicol and incubated at 37°C overnight.

### Screening for Higher Activity Mutants

Single colonies were picked and inoculated into 96-well plates containing 150 μl LB supplemented with 25 μg/ml of chloramphenicol and cultured at 37°C overnight. 20 μl of culture from each well was transferred into the corresponding well of a new 96-well plate, 80 μl of MOPS buffer (100 mM, pH 7.7) containing 5 mM KCN were added to each well and the plates were sealed with parafilm and incubated in a fume hood at room temperature for 20 min. The reaction was stopped by the addition of 100 μl picric acid solution (0.6% picric acid in 250 mM sodium carbonate) and plates were left at 60°C for 20 min. Under these conditions, cells carrying the wild-type *CynD* do not completely degrade all the cyanide present and the well retains a red or dark orange color. Any mutant with increased cyanide degrading activity was chosen if the cyanide in the well was completely degraded; in such case the well had a bright yellow color.

### Construction of Single, Double, and Triple Mutants

Alleles of CynD carrying single or double mutations were constructed using site-directed mutagenesis. The mutagenesis reactions were carried out with 25 μl Phusion High-Fidelity PCR Master Mix with HF Buffer (New England Biolabs), 150 ng dsDNA template and 100 ng of each of the forward and reverse primers. MQH_2_O was added to the reaction mixture for a total volume of 50 μl. Each reaction was run for 18 cycles at 93°C for 30 s, primer’s annealing temperature for 1 min and 72°C for 5 min. The mutagenic primers are listed in Supplementary Table S2. The mutations were confirmed by DNA sequencing.

### SDS-PAGE and Western Blot

Overnight cultures were diluted 1:100 in 3 ml LB kanamycin and grown to an OD_600_ of 0.3 at 37°C, at which point 1 mM IPTG was added for 3–4 h and induction continued at 30°C. Cells from 3 ml of induced culture were pelleted by centrifugation and washed twice with 0.1 M MOPS pH 7.7. The cell pellet was resuspended in 300 μl of 0.1 M MOPS pH 7.7 and sonicated on ice with 10 bursts of 10 s each using a microprobe. Cells debris was centrifuged and the supernatant was recovered and transferred to a new tube. The cell debris was also resuspended in 300 μl of 0.1 M MOPS pH 7.7. 10 μl of the supernatant or the resuspended cell debris suspension were mixed with 3 μl of Amresco 4X Next Gel Sample Loading Buffer, boiled for 10 min and run on an Amresco Pro-Pur Next Gel 10% polyacrylamide gel along with Ez-Run Prestained *Rec* Protein Ladder (Fisher Bioreagents). The gel was then electroblotted to a nitrocellulose membrane (Whatman) and anti-nitrilase polyclonal rabbit serum was used as primary antibody (1:3000) and HRP conjugated goat anti-rabbit IgG as the secondary antibody. SuperSignal West Pico rabbit IgG Chemiluminescent detection kit (Thermoscientific) was used to visualize the protein bands.

### Protein Expression and Purification

Wild type CynD and variants were cloned into the expression vector pET28a using NdeI and XhoI restriction sites and transformed into MB1837 (BL21 pLysS) cells. Protein was expressed as described in the previous section and the pellet was stored at -20°C overnight. Cells were thawed and lysed at room temperature with B-PER II Protein Extraction Reagent (Thermo Scientific) using 2 mL of B-PER II reagent per gram of cell pellet supplemented with 0.5 mg/ml lysozyme, 10 μg/ml DNase and 1X EDTA-free protease inhibitors as recommended by the manufacturer. The cell lysate was centrifuged at 13,000 rpm for 15 min at 4°C. Purification of hexahistidine-tagged proteins was performed at 4°C using the HisPur Cobalt Purification Kit (Thermo Scientific). Desalting of the purified protein at 4°C used Zeba Spin Desalting Columns (Thermo Scientific) and the protein was resuspended in 0.1 M MOPS pH 7.7. Protein concentration was determined using the NanoDrop ND-1000 at 280nm using the predicted his-tagged protein extinction coefficient of 58,790 cm^-1^ M^-1^ and molecular weight of 39,654 g/mol. SDS-PAGE confirmed the purity of the protein. Purified proteins were stored in aliquots at -80°C.

### Measurement of Enzyme Kinetics

3.9 μg of enzyme (9.84 × 10^-11^ moles of enzyme monomer) was used for kinetics analysis of wild type and CynD variants in a total volume of 1 ml and a final monomer concentration of 9.84 × 10^-5^ mM. A stock solution of 1 M of cyanide was prepared in 1 M MOPS pH 7.7 and reactions were done at room temperature in 50 mM MOPS at pH 7.7 at different concentrations of cyanide. Ammonia production was monitored by mixing 100 μl of reaction with 100 μl of diluted Nessler reagent (Nichols and Willits, 1934) (1:3 in MQ H_2_O) in a succession of wells in a 96 well plate. The absorbance of each well at 420 nm, which is proportional to the concentration of ammonia, was determined. Each reaction was run for 5 min and was sampled every minute. The change in absorbance over the first 5 min was linear enabling *v*_initial_ to be calculated. *K*_m_ and *V*_max_ were calculated using the Enzyme Kinetics plugin of Sigmaplot (Systat Software) by non-linear regression. For each enzyme variant, the quoted *K*_m_ and *V*_max_ values are averages of results from three separate protein preparations. *V*_max_ was used to calculate *k*_cat_ assuming that every monomer with a molecular mass of 39654 g/mole was active. Previous work (Jandhyala et al., 2003) suggests that the terminal monomers in the eighteen monomer configuration that exists at pH 7.7 may be inactive.

### pH Activity Measurement

5.5 μg of enzyme was used for pH activity analysis of the wild type enzyme and CynD variants in a total volume of 1 ml using protein purified as described above. Three cyanide stock solutions were prepared in 1 M citric acid/Na_2_HPO_4_ at pH 5.5, 1 M MOPS pH 7.7, and 1 M CAPS at pH 9.5. Reactions were done at room temperature in a fume hood at a final concentration of 4 mM cyanide. The activity of the purified enzyme was measured over the pH range 4–10. Buffers used were as follows: 50 mM citric acid/ Na_2_HPO_4_ (pH 4, 5, and 6), 50 mM MOPS (pH 7), 50 mM Tris-HCl (pH 8) and 50 mM glycine/NaOH (pH 9 and 9.5). The ammonia concentration was measured every minute for the first 5 min as described above. Final values are averages of results from three separate protein preparations.

### Enzyme Stability

The thermostability of each enzyme was determined by incubating 80 μg of purified protein in a total volume of 8 ml of 50 mM MOPS pH 7.7 in a water bath at 42°C using protein purified as described above. For each time point 0.5 ml of mixture was removed from 42°C and placed into a new 1.5 ml microfuge tube and left at room temperature for 3 min to equilibrate before adding cyanide in 1 M MOPS pH 7.7 to a final concentration of 4 mM. The reaction continued at room temperature and the activity was monitored for the first 5 min with time points taken every min to measure ammonia production as described above. The reaction rate was calculated using the zero time value as 100% and the relative activity of the enzyme at the different incubation times was then calculated. Final values are averages from three separate protein preparations.

### Calculation of ΔΔG^∗^

A quantitative estimate of the thermostability of the variants were obtained by calculating the differences in the activation energy for thermally induced activity loss between the wild-type CynD and the variant (ΔΔG^∗^) using the data from above. The rate of thermal inactivation, *k*_d_, was obtained for both the wild-type CynDpum and the variant enzymes by non-linear regression analysis in which the equation (% residual activity) = a. exp(-*k*_d_. t), where t is the time of incubation, was iteratively modeled using Sigmaplot. The observed deactivation rate constant, *k*_d_, can be related to ΔG^∗^, the activation energy, by the Arrhenius equation:

$${\text{k}}_{\text{d}}\;\text{=}\;\text{A}{\text{e}}^{\text{-}(\Delta {\text{G}}^{\text{*}}\text{/RT})}$$

where R is the gas constant and T is the absolute temperature at which the experiment was done. Hence ΔΔG^∗^ = ΔG^∗^_wt_ – ΔG^∗^_mut_ = RT ln (*k*_wt_/*k*_mut_). Positive values indicate that the variant is more thermostable than the wild-type enzyme.

### Construction of a Homology Model

The sequence of CynD was aligned to that of the Nit6803 using the UCSF chimera sequence plugin. A series of 10 homology models were created using the crystal structure PDB ID:3WUY as a template with MODELLER version 9.14 (Sali and Blundell, 1993) within this plugin and the one with the lowest zDOPE score was chosen.

## Results

### Screening for Variants with Higher Cyanide Degrading Activity at pH 7.7

The aim of this study was to isolate CynD variants with improved activity at the optimal pH of 7.7. Randomly mutagenized *CynD* DNA was generated by error-prone PCR followed by *in vivo* cloning to create the mutant library. Using 96-well plates and a colorimetric assay to estimate cyanide levels, approximately 5000 highly mutagenized clones were screened. The condition for the screen was chosen as one-half the reaction time needed by wild-type CynD expressing cells to completely degrade the cyanide, visualized by a color change from red (cyanide remaining) to yellow (cyanide absent) in the picric acid assay (Fisher and Brown, 1952). Putative mutants with increased activity showed a yellow color due to the complete degradation of cyanide whereas the wild-type parent remained red since the cyanide was only partially degraded. The increased activity of the mutants selected was confirmed by repeating the assay in a more accurate and reproducible sealed tube assay. Three mutants (CD12, DD3, and 7G8) from the total of about 5000 screened showed higher activity at pH 7.7.

### Characterization of the High Activity Mutants

DNA sequencing of the plasmids revealed that CD12 had one mutation resulting in the amino acid change E327K and 2 silent mutations (T849C and T957A). DD3 had one mutation resulting in the amino acid change K93R and one silent mutation (A867G). 7G8 had two mutations resulting in the amino acid changes D172N and A202T and one silent mutation (T570C).

Plasmids capable of expressing protein variants having each of the four single amino acid point mutations, the double mutant pairs K93R/E327K, K93R/A202T, E323K/A202T, D172N/A202T, and the triple mutant K93R/D172N/E327K were reconstructed in the absence of the silent changes using site-directed mutagenesis and were used for all subsequent analysis.

No CynD activity was detected in the case of the *E. coli* expressing the additional protein variants carrying the A202T mutation. Thus only D172N was able to compensate for the effects of A202T and not K93R nor E323K. To check whether protein variants carrying the A202T mutation were expressed but inactive or merely failed to accumulate in the cell, soluble protein extracts from induced cultures grown to similar OD_600_ were separated by SDS-PAGE and the CynD protein detected by western blotting as described in Materials and Methods (Supplementary Figure S1A). The 37 KDa CynD protein is readily detected on the blot and is absent in the negative control lane. All active mutants expressed levels of protein that were similar to that of the wild type plasmid. However, the inactive A202T single mutant (Supplementary Figure S1A) was barely detectable in the soluble cell lysate (Supplementary Figure S1A, lane 6).

To analyze the fate of the A202T protein, the cell pellet was solubilized in loading buffer, separated by SDS-PAGE and the presence of CynD was assessed by Western blotting. The A202T and the inactive double mutants E327K/A202T and K93R/A202T were found predominantly in the pellet, whereas the active double mutant, D172N/A202T was found in the lysate (Supplementary Figure S1B). The presence of the mutants containing A202T in insoluble aggregates or inclusion bodies is thus the most likely explanation for their low activity. This phenotype appears to be rescued by D172N (Supplementary Figure S1B, lane 8) as the double mutant is both soluble and active.

### Measurement of the Activity of the Purified Enzyme Variants

Wild type CynD along with the mutants K93R, D172N, E327K, D172N/A202T, and K93R/D172N/E327K were expressed with a his-tag and purified. The rate of ammonia production (Nichols and Willits, 1934) was measured using 3.9 μg of each purified enzyme (9.84 × 10^-5^ mM of monomers) at cyanide concentration from 1 mM to 8 mM. The rate of reaction was found to be linear during the first 5 min.

**Table 1** summarizes the *K*_m_ and *V*_max_ values found for each allele (other than the inactive A202T single mutant). Each mutant had kinetic parameters that differed from the wild type. E327K and K93R had similar enzyme kinetics and showed a slight increase in *V*_max_ compared to wild type but their affinity for the substrate decreased by a factor of 2.

**Table 1:** Kinetic parameters for wild type CynD and variants.

Enzyme	*K*_m_ (mM)	*V*_max_ (mmole.min^-1^)	*k*_cat_ (min^-1^)	*k*_cat_/*K*_m_ (min^-1^mM^-1^)
WT CynD	3.6 ± 0.4	0.162 ± 0.008	1.65E + 06	4.55E + 05
K93R	7.9 ± 0.7	0.26 ± 0.01	2.66E + 06	3.36E+05
D172N	0.7 ± 0.2	0.059 ± 0.003	6.02E + 05	8.01E + 05
E327K	5.0 ± 1.0	0.20 ± 0.02	2.01E + 06	4.04E + 05
D172N/A202T	0.9 ± 0.3	0.047 ± 0.003	4.81E + 05	5.13E + 05
K93R/D172N/E327K	5.0 ± 1.1	0.114 ± 0.01	1.16E + 06	2.19E + 05

*The *K*_*m*_ and *V*_*max*_ values are the average and the standard deviation of the average from three different protein preparations.*

D172N had higher affinity for cyanide with a fivefold decrease in *K*_m_ compared to wild type but its maximal velocity was less than half. The D172N/A202T allele had a similar phenotype to D172N. Its *K*_m_ and *V*_max_ values were lower than wild-type and of E327K and K93R. This suggests that A202T mutation contributes little to the phenotype of the double mutant.

The triple mutant K93R/D172N/E327K combined the effects of the active single mutants. Its *K*_m_ is about the average value of that of the single mutants, making it similar to the parent enzyme. However, its maximal velocity was lower than the wild-type, similar to the *V*_max_ of the single mutant D172N (**Table 1**).

### Thermostability

**FIGURE 1:**
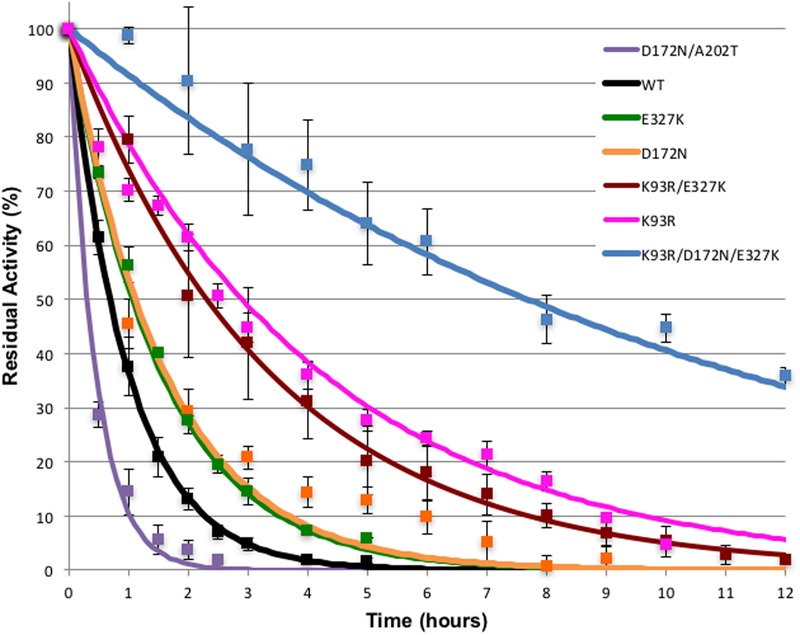
**Thermostability of CynD and variants.** Purified enzymes were incubated at 42°C for the times shown. Residual activity was tested at room temperature. Error bars indicate the standard deviation of the average from experiments conducted with three different protein preparations.

Thermostability has been used as a proxy for general stability with the cyanide degrading enzymes. To test if any mutants had an increase in stability, their thermostability was compared to that of the wild type enzyme. In a prior study (Jandhyala et al., 2005), the stability of CynD and CHT was tested by incubation at different temperatures and extended incubation at 42°C was found to be a suitable criterion. Therefore, each enzyme was incubated in a water bath at 42°C and at different time-points aliquots were removed and the activity remaining was assayed at room temperature. The cyanide degrading activity before incubation at 42°C was defined as 100% for each enzyme (**Figure 1**).

The effect of the mutations on enzyme stability varied. All three single mutations increased the stability of the enzyme. D172N and K93R (DD3) mutations had the highest effect on the enzyme stability (**Figure 1**). After 2 h incubation at 42°C, K93R, D172N, and E327K mutants retained 60, 30, and 30% of their residual activity, respectively, compared with the wild type which retained only 10% of its activity. The K93R/E327K double mutant was comparable to E327K. The triple mutant K93R/D172N/E327K was the most stable with 90% of its activity remaining after 2 h incubation at 42°C. This additive effect of the single mutations suggests a synergistic interaction, thus the various mutations are likely to affect the enzyme in different ways.

On the other hand, the double mutant D172N/A202T (7G8) had an unanticipated phenotype. It was less stable than the wild-type and the D172N single mutant. This is likely explained by the fact that single mutation A202T rendered the enzyme inactive. As shown previously on the western blot (Supplementary Figure S1B), A202T was insoluble and formed inclusion bodies or aggregates. Thus, it can be suggested that when the highly stable D172N mutant is combined with single mutation A202T (the 7G8 mutant), it partially repairs the inactivity of A202T mutant by compensating for the decrease in stability thereby retaining catalytic activity. The isolation of these two combined in the G78 mutant was likely coincidental.

The data in **Figure 1** was used to calculated the ΔΔG^∗^ of the mutants, shown in **Table 2** for comparison. All mutants except for the D172N/A202 showed a significant increase in thermostability with the triple mutant being dramatically more thermostable. The contributions of the individual mutations to the thermostability of the variants containing double and triple mutations are cumulative.

**Table 2:** Thermostability of the CynD variants relative to wild-type.

Variant	*k*_d_ (h^-1^)	α (*k*_d_)	ΔΔG^∗^ (kJ/mol)
WT	1.0111	0.0193	-
E327K	0.6259	0.0211	1.26
D172N/A202T	2.2447	0.1533	-2.09
K93R	0.2389	0.0304	3.78
D172N	0.6539	0.0772	1.14
K93R/E327K	0.2997	0.0187	3.19
K93R/D172N/E327K	0.0902	0.0295	6.33

*α(*k*_*d*_) indicates the standard error of the regression analysis.*

### pH Activity Profile

**FIGURE 2:**
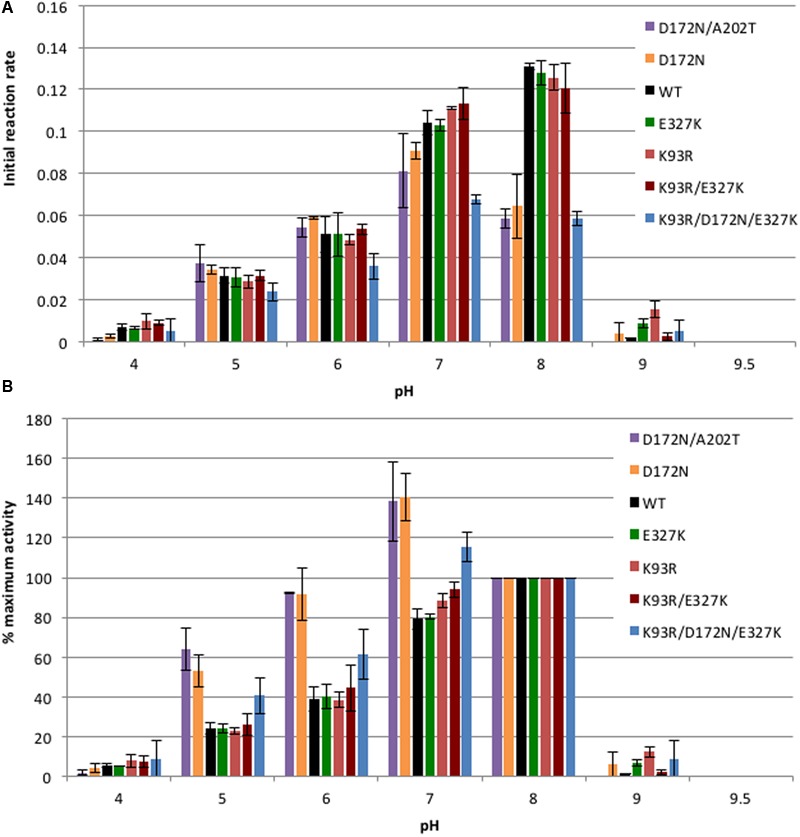
**Comparison of pH activity profile. (A)** 5.5 μg/ml of enzyme was tested with 4 mM cyanide and activity was measured at each pH. Initial reaction rate is represented by the change in absorbance (ΔA420/min) of the product formed by the reaction of ammonia and Nessler’s reagent at 420 nm per minute of the enzymatically catalyzed reaction. All enzyme variants tested were inactive at pH 9.5. Error bars indicate the standard deviation of the average from experiments conducted with three separate protein preps. **(B)** Data re-plotted to show percentage of activity at each pH relative to the activity at pH 8. The initial reaction rate at pH 8 of each enzyme was taken as 100%.

In previous work we showed that wild-type CynD had its maximal activity in the pH range of 7–8 with a peak around pH 7.7, at higher pH the enzyme sharply lost activity (Jandhyala et al., 2005). We also identified higher stability mutants that showed an increase in cyanide degrading activity at pH 9 (Wang et al., 2012). No significant increase in activity at pH 9 was observed for any of the purified mutant enzymes compared to the wild-type CynD (**Figure 2**). The increased thermal stability of the enzyme did not compensate for its lack of activity *in vitro* at alkaline pH. None of the mutants had any activity when tested at pH 9.5.

The activity of the enzyme at different pHs was plotted two different ways in order to clearly show the effect of the mutations. **Figure 2A** shows the initial reaction rate expressed in terms of absorbance of the product measured at 420 nm generated per min. This graph allows for a comparison of the actual activity of the enzymes at each pH while **Figure 2B** shows the percentage of activity for each enzyme at each pH relative to its activity at pH 8. Single mutants E327K and K93R as well as the double mutant K93R/E327K exhibited a similar pH activity profile to the wild-type with the maximal activity around pH 8. On the other hand, D172N, D172N/A202T, and K93R/D172N/A202T showed lower activity than wild type at pH 7 and especially at pH 8 (**Figure 2A**). Mutants carrying D172N had a lower *V*_max_ than wild-type at pH 7.7 (**Table 1**). Thus, the decrease in activity seen could be the result of the decrease in *V*_max_ value and is likely the contribution of the D172N change common to these three.

Since the wild-type showed a maximal activity at pH 8, the relative activity of each enzyme was plotted with the initial reaction rate at pH 8 taken as 100% activity (**Figure 2B**). It is clear that enzymes carrying the D172N mutation exhibited a shift in their optimal activity from pH 8 to pH 7. The D172N/A202T double mutant and D172N single mutant exhibited very similar pH activity profiles which support the idea that A202T has little effect. Nearly a 30% increase was noted in their activity at pH 7 compared to pH 8 and at pH 6 the mutant retained more activity than the wild-type enzyme, unlike E327K, K93R, and K93R/E327K which lost activity similar to wild type.

### Homology Model

**FIGURE 3:**
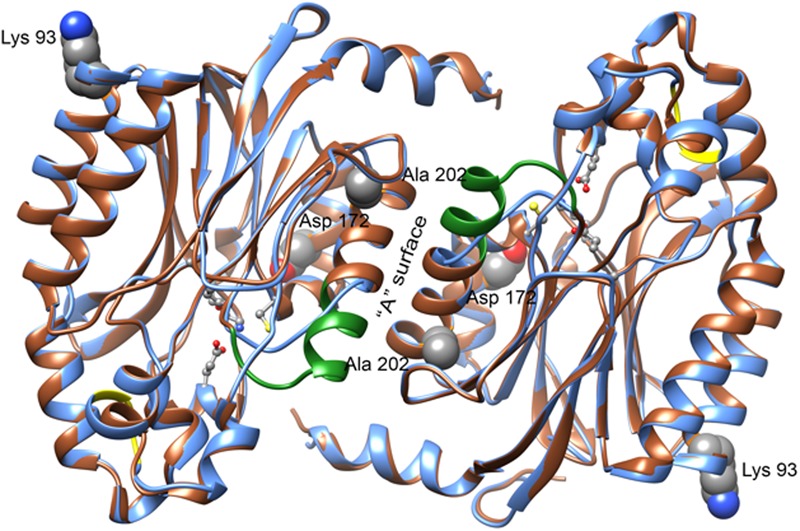
**Features of the CynD model obtained by homology with the structure of Nit6803 (PDB: 3WUY) (Zhang et al., 2014).** A ribbon diagram showing a superposition of the residues of Nit6803 that were visible in the crystal structure (brown) and the corresponding model of CynD (blue). Two monomers in contact across the “A” surface are depicted as they are seen in the crystal structure. The residues that are deleted in CynD relative to Nit6803 are shown in green and those that are inserted are shown in yellow. Four active site residues (Glu 48, Lys 130, Glu 137, and Cys 164) are shown as balls and sticks. Three of the residues discussed in this paper: Lys 93, Asp 172, and Ala 202 are shown as spheres. Ala 202 is located at the “A” surface, Asp 172 is directed toward the active site and Lys 93 is located near an interface in the crystal structure of Nit6803 that may be preserved in the CynD quaternary structure. The residue equivalent to Glu 327 was absent in the crystalline Nit6803.

The sequence of CynD has 31% identity to that of Nit6803. Our alignment (Supplementary Figure S2) shows that relative to the 286 residues located in the 3WUY (Nit6803) crystal structure CynD has a single insertion of two residues near residue 224 and a single deletion of 7 residues near residue 190 (CynD numbering). The deletion results in shortening the α-helix that forms the “A” contact surface between the monomers. This alignment was used to aid in building the model shown in **Figure 3**.

## Discussion

Using random mutagenesis followed by *in vivo* activity screening, we identified three *B. pumilus* CynD mutants that converted cyanide at pH 7.7 at a higher rate than *E. coli* expressing the wild-type enzyme. The mutants CD12 and DD3 had one amino acid change each, E327K and K93R, respectively, while 7G8 had two mutations D172N and A202T. Kinetics measurements, thermostability and pH activity profiles were used to characterize the effect of these mutations on enzyme activity and stability. These results in combination with structural modeling of the location of the residues help us suggest the role of these residues and better understand the CynD structure.

The wild-type CynD and all the mutants discussed form truncated spirals at neutral pH that elongate to form extended helices at pH5.4. An atomic model of *B. pumilus* CynD based on negative stain electron microscopy together with modeling of its sequence to those of related crystallographically determined structures (Jandhyala et al., 2003, 2005; Sewell et al., 2005) shows that the building block of the helix is a dimer of 39 KDa monomers. At pH 8, the wild-type enzyme forms an 18-subunit spiral with twofold symmetry. Four surfaces, previously designated A, C, D, and E as well as the C-terminus are predicted to be involved in interfacial interactions and proposed to play a role in protein oligomerization (Sewell et al., 2005) (Thuku et al., 2009). It is notable that the residues identified in this study K93R, D172N, A202T, and E327K are located on these important surfaces and their putative role is discussed below (**Figure 3**).

### A Surface Mutants: D172N and A202T

The proposed model of CynD suggests that the elongation of the spiral arises from the interactions at the A dimerization surface and C oligomerization surface. The A surface is formed by two α helices, α5 and α6, and interactions across this surface are important for connecting the monomer subunits. It was suggested that they also play a role in positioning the catalytic residue C164 in the active site (Kumaran et al., 2003). Mutations at this interface lead to loss of activity (Sewell et al., 2005).

A202 is highly conserved in the nitrilase superfamily. The A202T mutant is inactive, similar to other A surface mutations such as Y201D/A204D in the α6 helix and the A surface deletion Δ219–233 (Sewell et al., 2005). The majority of the A202T protein was found aggregated in the pellet indicating the possibility of abnormal folding (Supplementary Figure S1B). If α6 is crucial for dimerization and A202 has a crucial role on this surface, a threonine at this position may disrupt critical interactions and destabilize the dimer. This idea is supported by the restoration of activity when A202T was introduced with D172N, a mutant with strongly increased stability (**Figure 1**). The other mutations that had enhanced activity, K93R and E327K, did not restore function to the CynD harboring the A202T mutant. However, these residues are distant from the A surface lending further credibility to the notion that steric effects induced by D172N compensate for the deleterious effect of A202T.

Conversely, CynD harboring D172N alone, located on the α5 helix of the A surface, retained full activity. Its affinity for the substrate at pH 7.7 was fivefold higher but its maximal velocity was less than half (**Table 1**; **Figure 1**) of the wild type. This activity was higher at pH 7 than pH 8 by about 40% whereas the wild-type activity at this pH decreased by 20% (**Figure 2**). On the other hand, D172N led to a measurable change in the enzyme’s kinetics and, based on the proposed model for CynD, is in proximity of the active site (**Figure 3**). Previous suggestions that residues of α5 helps position the catalytic cysteine in the active site through strong hydrogen bonds (Kumaran et al., 2003) raises the possibility that D172N modified the affinity and the reaction rate by affecting the positioning of C164 residue, or other catalytic residues, in the active site.

### D/E Surface Mutant: K93R

Based on our modeling K93 is proposed to be located at the end of an α-helix (α3), that is distal to the “A” surface (**Figure 3**). This surface was designated the D/E surface (Sewell et al., 2005) signifying the putative different roles of this component of CynD at pH5.4 and neutral pH, respectively. Unlike the interactions at A and C surfaces that elongate the spiral, interactions at the E surface are thought to terminate the helix in the case of the 14 monomer spiral structure of CynD form *P. stutzeri* by tilting the last dimer and reducing the diameter of the spiral, thus preventing additional dimers from adding to the end of the spiral (Sewell et al., 2005).

The putative residues involved in this E surface interaction are the highly conserved negatively charged residues of the E2 region 267EID269 at the end of strand β14 (Jandhyala et al., 2003) and the positively charged residues of the E1 region 93KR94 on the carboxyl-terminal end of α3 (**Figure 3**).

As the pH is lowered to 5.4, CynD transforms to long fibers (Jandhyala et al., 2003), thus altering the conformation of the terminal monomer. It is tempting to speculate that replacing the lysine with the longer arginine side chain would change the dynamics of spiral formation and lock a larger number of subunits into the altered, active form. Support for this hypothesis is based on the phenotype of a different mutant, Q86R (Wang et al., 2012). Q86R created a more stable enzyme than the wild-type that at pH 9 formed elongated helices similar to the ones seen at pH 5.5. It was suggested that Q86R mutation resulted in an additional interaction at the D surface formed by a α1 and α3 thus increasing the stability of the helical oligomer.

Furthermore, Thuku et al. (2009) suggested that in the nitrilase of *Rhodococcus rhodochrous* J1, residue R94, which correspond to K93 in CynD, forms a salt bridge with residue D91 (E90 in CynD) across the D surface. Similar to the Q86 mutant, *R. rhodochrous* J1 nitrilase forms elongated helices. Thus the K93R mutation could actually be stabilizing the D surface with arginine increasing the hydrogen bonding with E90 and other negatively charged amino acids found at this surface. The explanation is, however, not straightforward as the similar *P. stutzeri* CynD that also has an arginine at this position does not form long helices but persists as a 14-subunit spiral. Further work on the structural consequences of the K93R mutation is needed to confirm our model.

### C-terminal Region Mutant: E327K

E327 is located in the carboxyl-terminal region, a highly variable extension of 30 to 60 amino acids found in the microbial nitrilases. No structural data of this region is available due to lack of sequence identity with related crystallographically determined structure. The nitrilases in the superfamily used for the protein docking lacked this extension and the low resolution cryo-electron microscopy failed to show clear detail. However, due to electron densities observed in the 3D reconstruction models, it was speculated that it is located in the center of the spiral. Studying the effects of various C-terminal truncations has led to clues on the role and location of this extension. Deletion of more than 28 amino acids from the *B. pumilus* CynD C-terminus disrupted its activity but shorter deletions remained active *in vivo* (Sewell et al., 2005; Crum et al., 2015a). Exchanging C-termini between the CynD enzymes from *B. pumilus* and *P. stutzeri* led to a dramatic increase in stability as well (Crum et al., 2015b).

Similarly *R. rhodochrous* J1 nitrilase lost activity when 55 C-terminal residues were deleted, however, cleavage of 39 amino acids from the tail is needed to form active oligomers (Thuku et al., 2007). Thus, it was speculated that this region might be interacting with the A and C surface and was important for the oligomerization of the protein.

*Bacillus pumilus* CynD at pH 8 formed short 18-subunit helices, however, at pH 5.5 extended spirals were seen and this effect was reversible (Jandhyala et al., 2003). It is believed that changes in side chain charges of histidines found in the carboxyl-terminus lead to an increase in helix length. At pH 5.5, three histidines, H305, H308, and H323 are protonated and would cause the tails to repulse disrupting the E surface and allow elongation (Abou Nader, 2012) of the spiral. Lysine substitution of these histidine residues increased the stability of wild-type (unpublished data from Mulelu). H323K which is in close proximity to E327, also increased overall stability. Based on the model, both residues H323 and E327 are located on the α11 helix. This indicates that E327 might be acting at the same surface, either A or C just like H323. The positive charge that the lysine brings stabilizes the surface similarly whether it is at position 323 or 327.

The E327K mutation increased the stability of the enzyme. However, its effect on stability was modest compared to that of K93R and D172N. E327 was also identified in a previous study, the mutant E327G increasing the pH tolerance of the nitrilase *in vivo* (Wang et al., 2012). In that case, the change was from glutamic acid, a negatively charged amino acid to glycine, a hydrophobic residue. This pH tolerance is thought to be a result of the enzyme’s stabilization at this alkaline pH. A similar phenotype was noted for E327K *in vivo* where it degraded cyanide at pH 9.

In this study, we characterized the K93R, D172N, A202T, and E327K mutations of CynD and described their effects on the enzyme’s characteristics from increasing the affinity to the substrate to increasing the *V*_max_ as well as on stability. Given the different mechanisms of action of these mutants, and their various locations in the protein, it is no surprise at synergy found in the constructed triple mutants K93R-A202T-E327K which was significantly improved over each individual mutant.

From the results, it is clear that interactions at the D/E and A surfaces as well as the C-terminal extension are important for enzyme stability, these surfaces have all been implicated by modeling as being important in formation of the final the quaternary structure of the protein. These mutants support the role of these interfaces. A detailed model of CynD would enlighten the mechanism of this phenotype, but that remains elusive in the absence of a higher resolution structure.

## Author Contributions

MC: Designed and conducted experiments, wrote first draft of manuscript. BS: Discussed and designed experiments, revised manuscript. MB: Designed experiments and discussed results, revised manuscript.

## Conflict of Interest Statement

The authors declare that the research was conducted in the absence of any commercial or financial relationships that could be construed as a potential conflict of interest.
