# Supplementary material for: Bacillus pumilus Cyanide Dihydratase Mutants with Higher Catalytic Activity
Source: Front Microbiol. 2016 Aug 12;7:1264. doi: 10.3389/fmicb.2016.01264 (PMC4981594; doi:10.3389/fmicb.2016.01264)
Supplement: Supplementary file 1 [file Data_Sheet_1.PDF]

Supplemental Materials

Bacillus Pumilus **Cyanide Dihydratase Mutant with Higher Catalytic Activity**

Mary A. Crum[1], B. Trevor Sewell[2] and Michael J. Benedik[1]*

[1]Department of Biology, Texas A&M University, College Station, TX 77843-3258, U.S.A.

[2]Structural Biology Research Unit, Department of Integrative Biomedical Sciences, University of Cape Town, South Africa

*Correspondence:

Michael J. Benedik
tel: (+1) 979-845-5776
fax: (+1) 979-845-2891
email: benedik@tamu.edu

Table S1. Plasmids used and constructed carrying various CynD alleles.

Plasmids	Description	Reference
pBS (KS+)	Cloning vector (Amp-r)	Stratagene
pBC (SK+)	Cloning vector (Cam-r)	Stratagene
pET26b	T7 expression vector (Kan-r)	Novagen
pET28a	T7 expression vector (Kan-r)	Novagen
pMB3980	pBS carrying *B. pumilus* CynD with N-terminal His-tag	(Wang *et al.*, 2012)
pMB4105	pBC-c*cdB*	(Abou-Nader & Benedik, 2010)
pMB4165	pMB4105 carrying CynD CD12	This work
pMB4240	pMB4105 carrying CynD 7G8with N-terminal His-tag	This work
pMB4606	pMB4105 carrying CynD DD3 with N-terminal His-tag	This work
pMB4257	pET26b carrying CynD CD12 with N-terminal His-tag	This work
pMB4265	pET26b carrying CynD 7G8 with N-terminal His-tag	This work
pMB4631	pET28a carrying CynD DD3 with N-terminal His-tag	This work
pMB4406	CynD from pMB3980 in pET28a	This work
pMB5466	pMB4406 E327K	This work
pMB5471	pMB4406 K93R	This work
pMB5472	pMB4406 A202T	This work
pMB5557	pMB4406 D172N	This work
pMB5591	pMB4406 D172N A202T	This work
pMB5496	pMB4406 K93R E327K	This work
pMB5593	pMB4406 K93R D172N E327K	This work
pMB5630	pMB4406 E327K A202T	This work
pMB5631	pMB4406 K93R A202T	This work

Table S2. Primer sequences used in site-directed mutagenesis.

Primer	Sequence
K93R	ATAAGTGAGGCAGCGCGCAGAAATGAAACGTAC
D172N	CAAGTCCCACTTAATCTTATGGCGATGAAT
A202T	CAAGTAGATATTATACTATAGCGACACAGAC
E327K	ATCAACATGGTATACTTAAGGAAAAAGTTTAA

Forward primer sequences. The underlined nucleotides indicate the substituted site.

Figure Legends

Fig S1. Western blot showing protein levels of CynD wild-type and mutants.

Top panel showing soluble cell extracts. Lane1 (from left): EZ-Run Prestained *Rec* Protein Ladder; lane 2: pET26b empty vector (negative control); lane 3: wild-type CynD; lane 4: K93R; lane 5: D172N; lane 6: A202T; lane 7: E327K; lane 8: D172N/A202T; lane 9: K93R/E327K; lane 10: K93R/D172N/E327K.

Lower panel showing protein levels of A202T CynD variants. Lane1: EZ-Run Prestained *Rec* Protein Ladder; lane 2: supernatant of pET26b empty vector (negative control); lane 3: A202T supernatant; lane 4: D172N/A202T supernatant; lane 5: E327K/A202T supernatant; lane 6: K93R/A202T supernatant; lane 7: pET26b pellet; lane 8: A202T pellet; lane 9: E327K/A202T pellet; lane 10: K93R/A202T pellet.

Fig S2. Alignment of B. pumilus CynD with Nit6803 (3WUY) used to generate the homology model described.

Figure S1

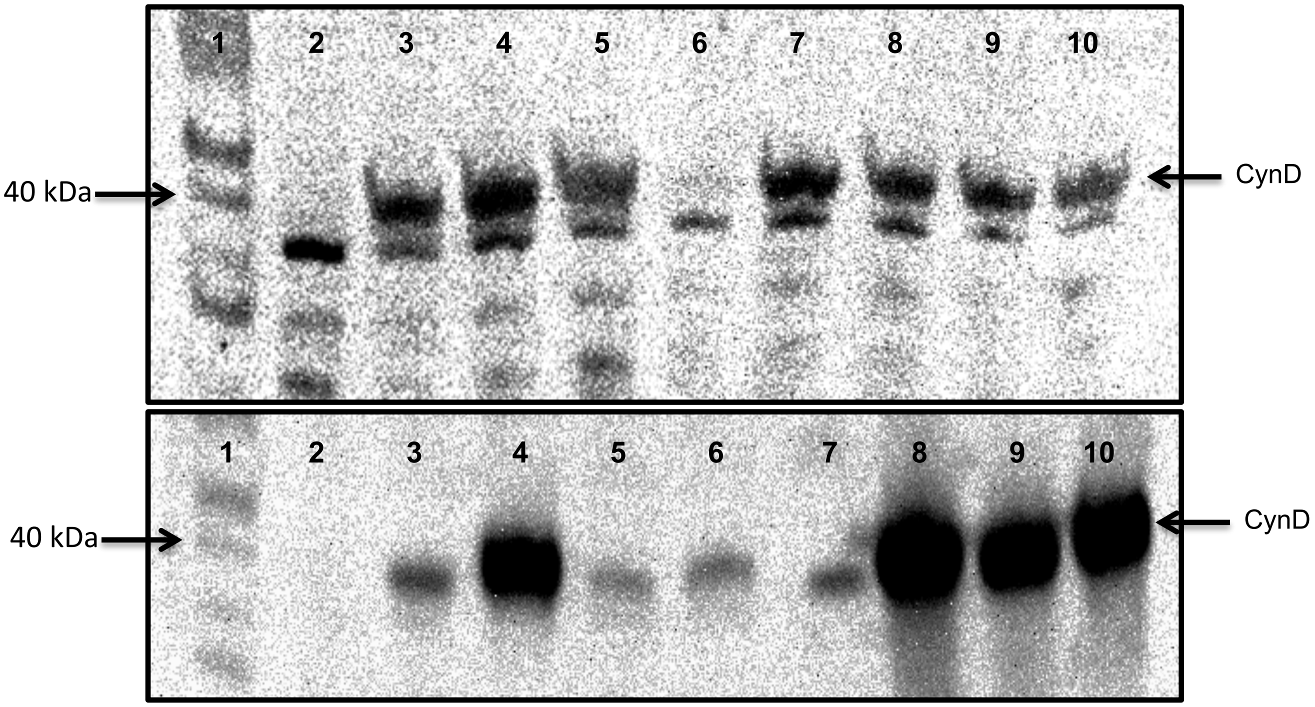

Figure S2.

```
Nit6803(3WUY).          .         .         .         .         .
 -2 GSHMLGKIMLNYTKNIRAAAAQISPVLFSQQGTMEKVLDAIANAAKKGVELIVFPETFVP 57
  1 --------MTSIYPKFRAAAVQAAPIYLNLEASVEKSCELIDEAASNGAKLVAFPEAFLP 52
CynDpum

              .         .         .         .         .         .
 58 YYPYFSFVEPPVLMGKSHLKLYQEAVTVPGKVTQAIAQAAKTHGMVVVLGVNEREEGSLY 117
 53 GYPWFAFIGHPEYTRKFYHELYKNAVEIPSLAIQKISEAAKRNETYVCISCSEKDGGSLY 112

              .         .         .         .         .         .
118 NTQLIFDADGALVLKRRKITPTYHERMVWGQGDGAGLRTVDTTVGRLGALACWEHYNPLA 177
113 LAQLWFNPNGDLIGKHRKMRASVAERLIWGDGSGSMMPVFQTEIGNLGGLMCWEHQVPLD 172

              .         .         .         .         .         .
178 RYALMAQHEQIHCGQFPGSMVGQIFADQMEVTMRHHALESGCFVINATGWLTAEQKLQI- 236
173 LMAMNAQNEQVHVASWPG-----YFDD--EISSRYYAIATQTFVLMTSSIYTEEMKEMIC 225

              .         .         .         .         .         .
237 -TTDEKMHQALSGGCYTAIISPEGKHLCEPI-AEGEGLAIADLDFSLIAKRKRMMDSVGH 294
226 LTQEQRDYFETFKSGHTCIYGPDGEPISDMVPAETEGIAYAEIDVERVIDYKYYIDPAGH 285

              .         .         .         .         .         .
295 YARPDLLQLTLNNQPWSALEANPVTPNAIPAVSDPELTETIEALPNNPIFSH         356
286 YSNQSLSMNFNQQPTPVVKHLNHQKNEVFTYEDIQYQHGILEEKV                330
```
